# Associations between perceived institutional support, job enjoyment, and intentions to work in the United Kingdom: national questionnaire survey of first year doctors

**DOI:** 10.1186/s12909-016-0673-6

**Published:** 2016-05-23

**Authors:** Shelly Lachish, Michael J. Goldacre, Trevor Lambert

**Affiliations:** UK Medical Careers Research Group, Nuffield Department of Population Health, University of Oxford, Old Road Campus, Old Road, Oxford, OX7 3LF UK

**Keywords:** Institutional support, Job enjoyment, Attitudes, Junior doctors, United Kingdom

## Abstract

**Background:**

Identifying factors that improve job satisfaction of new doctors and ease the difficult transition from student to doctor is of great interest to public health agencies. Studies to date have focused primarily on the value of changes to medical school curricula and induction processes in this regard, but have overlooked the extent to which institutional support can influence new doctors’ enjoyment of and attitude to work. Here, we examine variation in the perceived level of support received by new medical graduates in the United Kingdom (UK) from their employer and whether this influences enjoyment of and attitudes to the first postgraduate year, and whether doctors who perceived a lower level of support were less inclined to intend a long term career in medicine in the UK.

**Methods:**

All UK medical graduates of 2012 were surveyed in 2013 in a cross-sectional study, towards the end of their first post-graduate year (the ‘F1’ year of the 2-year Foundation Training Programme for new UK doctors). We used linear regression to assess whether the level of support doctors reported receiving from their employing Trust (*Very Good, Good, Adequate, Poor,* or *Very Poor)* was associated with the extent to which they enjoyed their F1 year. Similarly, we assessed the strength of associations between self-reported level of Trust support and doctors’ responses to 12 statements about fundamental aspects of their working lives, each assessed on a 5-point scale of agreement. Using *χ*^2^ tests we examined whether doctors’ intentions to practise medicine in the UK varied with the level of support they reported receiving from their Trust.

**Results:**

The response rate was 45 % (2324/5171). Of 2324 responding junior doctors, 63.8 % reported receiving ‘*Very Good*’ (23.6 %) or ‘*Good*’ (40.2 %) initial support from their Trust, while a further 27.4 % stated they received ‘*Adequate*’ support. *‘Poor’* support was reported by 5.8 % and *‘Very Poor’* support by 2.2 %. We found very strong positive associations between the institutional support doctors reported receiving and their enjoyment of the F1 year and their self-expressed attitudes to aspects of their first year of work. Crucially, doctors who reported receiving lower levels of support (‘*Poor*’ or *‘Very Poor’*) were significantly less likely to express intentions to continue practising medicine in the UK.

**Conclusions:**

The provision of effective institutional support for graduate doctors may promote workplace satisfaction and could help safeguard the long-term retention of junior doctors.

**Electronic supplementary material:**

The online version of this article (doi:10.1186/s12909-016-0673-6) contains supplementary material, which is available to authorized users.

## Background

The transition from medical student to junior doctor is a demanding and stressful period for many graduates [1]. The pressures of being new to a job, heavy workloads, intense emotional burdens, fear of litigation, and general feelings of ‘unpreparedness’ can lower morale and job satisfaction [2]. Research shows that job satisfaction is associated with employee retention rates in numerous work sectors [3, 4], including in health care [2]. More importantly, research shows that initial experiences within the first few months of starting work strongly influence career decisions [3]. Indeed, a British Medical Association study revealed that junior doctors’ desires to continue practising medicine fall in the first year of training [5]. Hence, for government agencies tasked with providing an efficient public health care service, it is vital to identify factors that improve the job satisfaction of first year doctors and facilitate a smooth transition from student to doctor.

The bulk of research conducted to date suggests that the transition from student to doctor may be eased by implementing strategic changes to medical school curricula and more effective induction processes at the onset of work [6–8]. In the UK, following directives for learning and competencies outlined in the General Medical Council’s *Tomorrow’s Doctors* reports [9, 10], undergraduate medical school curricula were revised to incorporate more integrated clinical learning and greater opportunities to experience relevant tasks (e.g. via student assistantships and ‘shadowing’ – that is, working for a time alongside a qualified postgraduate doctor as a learning experience prior to qualification). Some studies have reported that these changes improved the proficiency and the performance of new doctors [7, 11–13]. However, other studies have revealed continued shortfalls in skills and ‘preparedness’ for work, from the perspective of both graduates and their supervisors [8, 13–17].

Currently in the UK, all new medical graduates must complete a structured two-year Foundation Training Programme (termed the F1 and F2 years) prior to entering a core, specialty or general practice training programme. In the F1 year, medical graduates begin to take supervised responsibility for patient care and consolidate skills learned at medical school. In the F2 year, doctors continue to develop their core generic skills under clinical supervision but take on increasing responsibility for patient care. In particular, F2 doctors begin to make management decisions as part of their progress towards independent practice and contribute to the education and training of nurses, medical students and less experienced doctors.

The provision of posts and training during the Foundation Programme is coordinated at a regional level by ‘virtual’ bodies called Foundation Schools, which comprise the doctors’ employers (known as Health Boards in Scotland and Medical Trusts elsewhere in the UK, and which we refer to as Trusts throughout) and the educational providers (known as local education training boards [LETBs] in England and Deaneries elsewhere in the UK). New F1 doctors undertake short (typically 2–4 days) generic educational inductions from their LETB/Deanery prior to the commencement of work, designed to familiarise them with the clinical and non-clinical practices of their first job and involving a period of ‘shadowing’ (which became mandatory in 2013; [18]). In addition, junior doctors receive short (typically ≤1 day,) practical inductions from their respective Trusts (hospitals and departments) focusing on the information needed for working in their particular location [18, 19]. These short inductions have been criticised for being both insufficient and inefficient [20, 21]. While there is some indication that longer, more structured induction processes can offer greater improvements in clinical skills, competence and preparedness among new doctors [6, 8, 22], it is not clear whether such gains correlate with greater job satisfaction or increased morale.

In addition to curriculum changes at medical school and an effective and comprehensive induction process, support more broadly for graduate doctors in their work environment is also likely to ease their transition. In the UK, day to day, this responsibility for trainees feeling well-supported lies with the employing Trust. Trusts are tasked with ensuring new doctors understand the practicalities and logistics of working in that location (e.g. providing information on safety drills and procedures, how to request tests and obtain results, how to obtain a bleeper or pager), and ensuring that new doctors know their chain of supervision and how to access advice and resolve problems. They may also provide additional support for new doctors by offering mentoring schemes, ensuring that graduates receive good support from management, senior doctors and colleagues, and facilitating access to help and learning opportunities (but are not obliged to do so). As studies have shown that new doctors are often ill-prepared for the practicalities of clinical work [13, 17], the implementation of effective initial institutional support in these areas would be expected to ease these difficulties [23, 24]. Indeed, this was true for newly appointed hospital consultants [25]. Such support would also be expected to improve morale and job satisfaction amongst new staff.

Surprisingly, despite considerable interest in easing the student-doctor transition and enhancing morale across the health profession [23, 24, 26], few studies have yet examined whether the extent of overall institutional support that new doctors receive influences their enjoyment of and attitude to work [27]. In this study, we examine the evidence to support the hypothesis that self-reported job enjoyment and attitude to work of F1 doctors, including their level of intention to work long term in medicine in the UK, are positively associated with how much initial support the doctors reported receiving from their employing Trust.

## Methods

### Survey design

Since 1975, the UK Medical Careers Research Group has followed the careers of successive generations of UK doctors by conducting regular postal and, more recently, web questionnaire surveys. In this study, questionnaires were sent to doctors who had graduated from medicine in the UK in 2012, towards the end of their F1 year in 2013. Addresses were obtained for those doctors who had registered with the General Medical Council (GMC) and we sent up to three reminders to non-respondents. All respondents were also contacted by email and given the option to complete a web-based version of the survey that was identical in content. As in all our previous surveys, the questionnaire sent to the doctors was wide-ranging and multi-purpose, containing questions on career intentions, experiences of the F1 year, and attitudes to various aspects of their work and training.

We asked doctors to respond to the statement, ‘*The support provided by my employing Trust when I started my first F1 post was on the whole: Very Poor, Poor, Adequate, Good,* or *Very Good’*. Hence, doctors were specifically asked to evaluate the institutional support they received upon entering the medical workforce. As the type and extent of institutional support offered to new doctors varies greatly among the many employing Trusts, we did not further define the term ‘support’ for respondents, allowing them to self-define this term, and so capture their overall impression of the support they were offered by their particular Trust.

Doctors were also asked “*How much have you enjoyed the F1 year overall on a scale from 1 (didn’t enjoy it all) to 10 (enjoyed it greatly)?*”, and were asked to respond to the following 12 statements regarding aspects of their training and work, which we collectively view as ‘attitudes to work’ (on the questionnaire, statements on related topics were not listed consecutively as they are here):

1. *Training has been of a high standard*
2. *Educational opportunities have been good*
3. *I receive good support from senior doctors*
4. *I receive good support from nursing staff*
5. *I receive good support from management*
6. *I have found arrangements for my annual leave to be satisfactory so far*
7. *I have found cover for absent doctors to be satisfactory so far*
8. *I have got good cover from more senior doctors, when needed, outside normal working hours*
9. *I work longer hours than I think I should*
10. *I am currently under too much pressure whilst at work*
11. *I am expected to perform too much routine non-medical work*
12. *I had to perform clinical tasks for which I was inadequately trained*

These 12 attitude statements were developed based on common issues identified in other studies that have examined doctors’ views on work (e.g. [28, 29]) and augmented with themes raised in spontaneous comments we have received from doctors to our previous surveys [30]. For each statement doctors were asked to indicate their degree of agreement on a 5-point scale (*Strongly agree, Agree, Neither agree nor disagree, Disagree, Strongly disagree*). Finally, doctors were asked to respond to the question, “*Apart from temporary visits abroad, do you intend to practise medicine in the United Kingdom for the foreseeable future?*” Doctors could respond ‘*Definitely*’, ‘*Probably*’, ‘*Probably not*’, ‘*Definitely not*’ or ‘*Undecided*’.

### Statistical analyses

We used *χ*^2^ tests to examine whether the self-reported level of support received from the Trust varied between (a) men and women, (b) ethnic groups (aggregated by us into White and non-White, based on respondents’ descriptions of their ethnicity), (c) doctors who were graduate entrants to medical school or not, and (d) those who received their first choice of Foundation School or not. These *χ*^2^ tests were performed on three binary variables: (i) ‘*Very Good’* vs. all other responses, (ii) ‘*Very Good/Good’* vs. all other responses, and (iii) ‘*Very Poor/Poor’* vs. all other responses. We also used *χ*^2^ tests to examine whether doctors intentions to continue practising medicine in the UK (*Definitely/Probably*, *Probably not/Definitely not*, or *Undecided*) varied with the level of support they reported receiving from their Trust (the binary variables *ii, iii* described above).

We used linear regression to assess whether the level of initial support doctors reported receiving, treated as the independent variable, was associated with the extent to which doctors subsequently enjoyed their F1 year (using the enjoyment score between 1 and 10 described above, treated as the dependent variable). Linear regressions were also used to assess the strength of associations between self-reported level of initial support received from the Trust (from 1 = Very Poor to 5 = Very Good) and doctors’ attitudes to fundamental aspects of their working lives in the following year (as measured by the grouped median responses of doctors to each of the 12 statements listed above). We use a Bonferroni correction to adjust for multiple testing (*P* = 0.004).

## Results

Of the 5437 registered doctors who graduated in 2012, we were able to contact 5171, of whom 44.9 % (2324) responded to the questionnaire. The response rate among women was 48.1 % (*N* = 1463) and among men was 40.5 % (*N* = 861).

### Variation in the level of perceived support received from the Trust

While the vast majority of doctors (92 %) reported receiving at least *Adequate* support from their Trust in their first postgraduate year, far fewer (23.6 %) reported that the support they had received was *Very Good* (*Very Good* 23.6 % [*N* = 541]; *Good* 40.9 % [935]; *Adequate* 27.4 % [628]*, Poor* 5.8 % [133]*, Very Poor* 2.2 % [51]). There was no significant variation between men and women, between White and non-White doctors, between doctors who had been graduate-entrants to medical school and those who had not, or between doctors who did or did not get their first choice of Foundation School, in the level of support they reported receiving (Additional file 1: Table S1).

### The support of the Trust is associated with a greater enjoyment of and more positive attitude to the F1 year

**Fig. 1:**
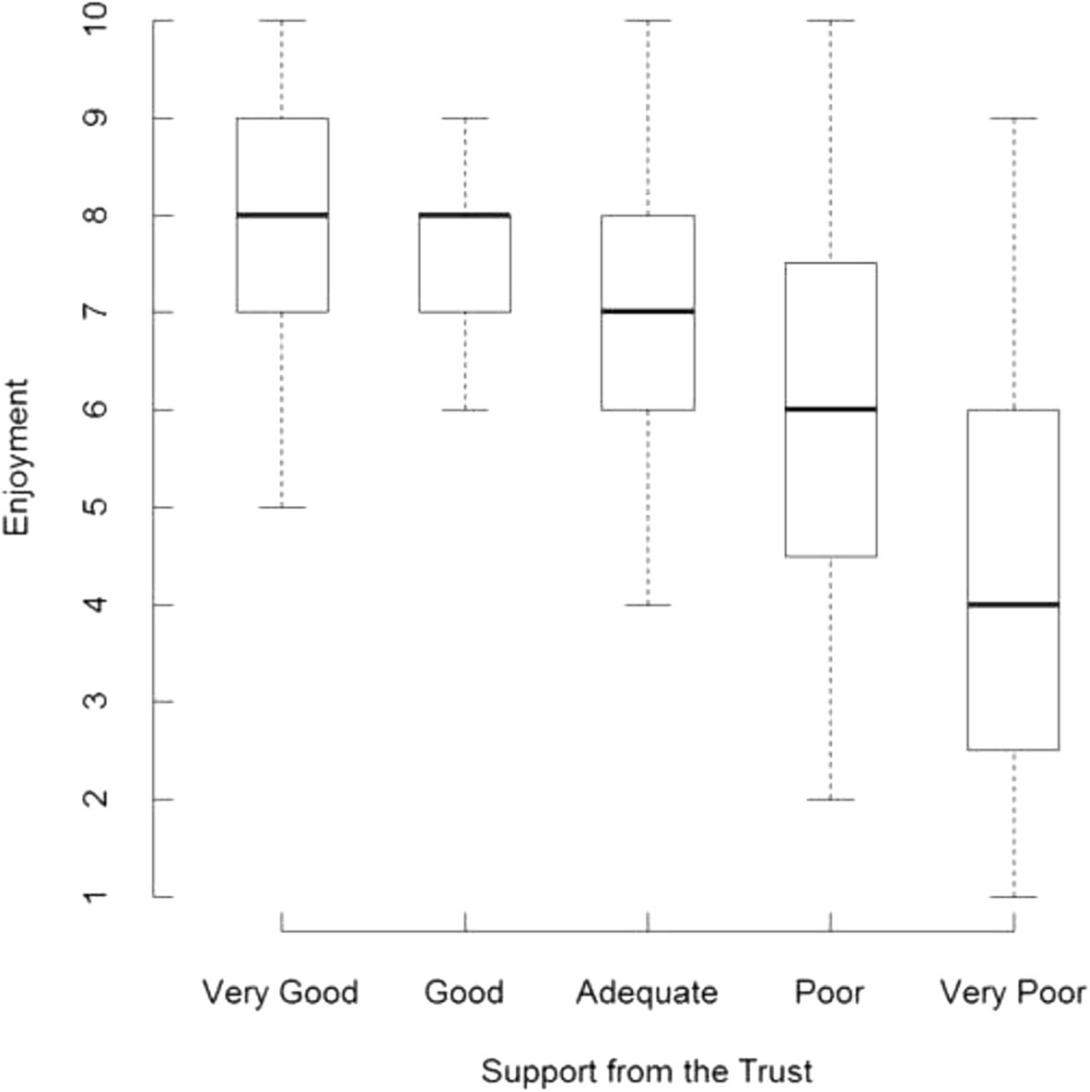
Association between support received from the Trust and doctors’ enjoyment of the F1 year

Doctors who received good support enjoyed their F1 year significantly more than did those who received poor support: the enjoyment score increased by 0.8 (on a scale from 1 to 10; *P* <0.001) for a one-step increase in support (Fig. 1). The median value of enjoyment of the F1 year expressed by doctors who reported receiving ‘*Very Good*’ support from their Trust was twice that of the doctors who received ‘Very Poor’ support (8 out 10, compared with 4 out of 10; Fig. 1). Doctors who felt well supported also responded in significantly more ‘positive’ terms to the majority of the 12 attitude statements than did doctors who reported that support from their school had been poor. Analyses revealed significant associations between perceptions of institutional support and eight of the 12 statements. Doctors more strongly agreed with the positive statements (statements 1, 2, 4, 5, 7) and more strongly disagreed with the negative statements (statements 9, 10, 11) as self-reported support from the Trust increased (Table 1). The level of support received from the Trust was most strongly associated with doctors’ attitudes towards training standards, educational opportunities and the support of management (statements 1, 2, and 5; Table 1). On average, doctors tended to agree with the positively-worded attitude statements (statements 1–8), and to disagree with the negatively-worded attitude statements (statements 9–12), though there were exceptions (statements 5, 7, 9, and 11; Additional file 2: Figure S1).

**Table 1:** Strength of association between support of the Trust and doctors’ responses to 12 attitude statements

Attitude statements	*Slope (Unstandardized betas with 95 % CI)* ^*a*^	*P*
1. *Training has been of a high standard.*	0.57^b^ (0.46, 0.66)	<0.001*
2. *Educational opportunities have been good.*	0.57 (0.45, 0.68)	<0.001*
3. *I receive good support from senior doctors.*	0.13 (0.05, 0.21)	0.016
4. *I receive good support from nursing staff.*	0.20 (0.12, 0.29)	0.004*
5. *I receive good support from management.*	0.49 (0.34, 0.64)	0.001*
6. I have found a*rrangements for my annual leave to be satisfactory so far.*	0.32 (0.03, 0.67)	0.064
7. I have found c*over for absent doctors to be satisfactory so far.*	0.28 (0.20, 0.36)	0.002*
8. *I have received good cover from senior doctors when working outside normal hours.*	0.17 (0.03, 0.38)	0.075
9. *I work longer hours than I think I should.*	−0.21 (−0.26, −0.16)	<0.001*
10. *I am currently under too much pressure whilst at work.*	−0.24 (−0.27, −0.21)	<0.001*
11. *I am expected to perform too much routine non-medical work.*	−0.30 (−0.30, −0.29)	<0.001*
12. *I had to perform clinical tasks for which I was inadequately trained.*	−0.06 (−0.15, 0.03)	0.126

^a^Associations are given as the slopes (betas and their 95 % CI) of relationships between the grouped median responses of doctors to each of the 12 attitude statements and the support received from the Trust (1 = Very Poor through 5 = Very Good), as estimated by least-squares linear regressions. All trends were close to linear, except for annual leave (statement 6), cover after hours (statement 8) and clinical tasks (statement 12)

^b^The slope parameter for this regression indicates that for a one unit increase in the level of perceived support from the Trust the (grouped median) level of agreement with this statement increases by 0.57 (on a scale from 1 to 5, where 1 = Strongly Disagree and 5 = Strongly Agree)

*Significant after Bonferroni correction (*P* =0.004)

### Support of the Trust and intentions to practise medicine in the UK

The majority of respondents (74 %) stated that they intended to practise medicine in the UK for the foreseeable future (‘*Definitely*’ = 34.7 %; ‘*Probably*’ = 39.6 %, ‘*Probably not*’ = 7.6 %; ‘*Definitely not*’ = 1.2 %, *Undecided* = 16.9 %). The percentage of doctors who intended to practise medicine in the UK (*Definitely/Probably*) was greater among those who received *Very Good/Good* (77 %) support than among those receiving less support (70 %; Table 2). Conversely, fewer doctors who reported receiving *Poor/Very Poor* support intended to continue practising medicine in the UK (65 %) than those who reported receiving better support (76 %; Table 2). Indeed, almost twice as many doctors who reported receiving *Poor/Very Poor* support did not intend (*Definitely not/Probably not*) to continue practising medicine in the UK (15 %) as those who felt better supported (8 %; Table 2). The percentage of doctors who were *Undecided* in this respect was also greater among those who received less support (*Poor/Very Poor*: 20 %) compared to those who felt better supported (15 %; Table 2).

**Table 2:** Associations between support of the Trust and doctors’ intentions to continue practising medicine in the UK^a^

Level of support	“*Do you intend to practise medicine in the UK for the foreseeable future?*”		
	Definitely/Probably	Undecided	Probably not/Definitely not
	% (N/Total)	% (N/Total)	% (N/Total)
*Very Good/Good* ^*b*^	77.2 (1137/1472)	14.8 (218/1472)	7.9 (117/1472)
All other responses	69.8 (565/810)	20.0 (162/810)	10.2 (83/810)
*Poor/Very Poor* ^*c*^	64.5 (118/183)	20.2 (37/183)	15.3 (28/183)
All other responses	75.5 (1584/2099)	16.2 (343/2099)	8.2 (172/2099)

^a^Denominators are the number of respondents per category

^b^ *χ* ^2^ = 15.53, df = 2, *P* <0.001; ^c^ *χ* ^2^ = 13.94, df = 2, *P* = 0.001

## Discussion

Based on the responses of F1 doctors who graduated in the UK in 2012, we have shown that the perceived level of institutional support initially offered to new doctors by their employing Trust is associated not only with greater job enjoyment and more positive attitudes to work, but also influences these doctors’ self-expressed intentions to continue practising medicine in the UK.

Comparing those who reported receiving *Very Good* and *Very Poor* initial support from their Trust, the former scored twice as highly as the latter in the extent to which they reported enjoying their first Foundation training year. Likewise, well-supported doctors viewed a range of work-related issues more positively than poorly-supported doctors. New doctors who felt that the initial support from their Trust had been poor were more likely to feel that training standards and educational opportunities were inadequate, that they did not receive good support from management and nursing staff, and that they felt overworked, under pressure, and overwhelmed by routine nonmedical work. Crucially, intentions to continue practising medicine in the UK were significantly lower amongst doctors who reported receiving lower levels of support from their Trust. This echoes a German study which found that junior doctors in a less structured residency program tended to have a greater intention to leave [31]. These important findings suggest that enhancing the degree of institutional support offered to new doctors in the early stages of their first year may be an effective means of increasing workplace satisfaction in new doctors and facilitating the transition from student to doctor.

The realisation that junior doctors need to be supported in the early stages of their first jobs is not new. Previous studies have shown that lack of organisational support can increase anxiety in junior doctors [5, 32], while strong support for junior doctors from senior doctors and peers can improve their wellbeing, reduce burnout [23], and increase their ability to cope with the demands and responsibilities of their position [33]. Nevertheless, there is also a clear recognition, particularly within the UK, that current levels of support for junior doctors could be greatly improved [34, 35]. A recent GMC report [36] revealed that trainee doctors often find it challenging to gain the support of seniors and feel least supported when working out of hours. However, clear agency-led directives detailing methods to strengthen and implement institutional support systems for all trainee doctors are lacking. In the *Rough Guide to the Foundation Programme*, the types of institutional support available to new trainees are described under the heading “What if things go wrong?” [18]. Our work suggests that there are substantial benefits to be reaped by providing strong institutional support to all new graduates proactively, rather than reactively as is indicated above.

In this study, low sample sizes per Trust precluded analysis of the variation in the level of Trust support among the many UK medical Trusts. Variation in the quality of supervision and teaching among medical institutions has, however, been noted previously by the GMC [37]. This has led to concerted efforts by the GMC to identify how and where to provide support to doctors, particularly in the initial stages of their Foundation course [38]. Our attempts to identify aspects of doctors’ work that were significantly associated with their perceptions of institutional support indicates that enriching training and education opportunities streamlining HR and administration processes, and ensuring strong support from management may be fruitful initial undertakings for institutions wishing to improve their support system for graduates. Certainly, strong relationships between junior doctors and hospital management underpin the success of quality improvement projects [39]. However, other aspects of institutional support offered to junior doctors, such as more comprehensive induction programmes, good childcare services, or novel mentoring programmes, may also strongly influence their attitude to and enjoyment of their work. Further work in this area, using a combination of targeted questionnaires and follow-up structured interviews, should aim to identify key areas of support that are most valuable to new graduates and most readily amenable to improvement by the agencies responsible. This undertaking will provide the basis for developing more effective institutional support systems for new trainee doctors. Moreover, ensuring institutional support facilitates and enhances training and learning experiences should also produce new doctors who are better-prepared for clinical practice [13].

We note that overall only 8 % of the doctors who responded to our survey reported receiving poor support from their Trust: more than 90 % reported receiving at least adequate support. These figures should be reassuring to those agencies responsible for ensuring the well-being of new doctors. There is, nevertheless, scope for improvement. We found that doctors’ enjoyment of the F1 year and ‘positivity’ towards many work-related issues increased linearly with increasing levels of perceived early institutional support. In addition, intentions to continue practising medicine were also markedly higher among doctors with higher perceived levels of institutional support. Although association does not prove causation, there may be important gains to be made in enhancing the well-being and morale of graduate doctors, even by improving institutional support levels from ‘Adequate’ to ‘Good’.

This study was conducted on a national scale and incorporates the views of more than 2300 junior doctors across the UK. As our surveys are conducted independently of any organisation that employs, trains, or influences the doctors’ careers, we believe we get honest answers from the respondents. We acknowledge, however, that self-reported perceptions of support may not reflect objective assessments of available support and our results should be interpreted in light of this possibility. Yet if institutional support structures do exist but doctors are unaware of them or unable to access them, then this also constitutes lack of effective institutional support. As we do not have a meaningful measure of differences in the support schemes offered to new doctors at Trusts, we can only speculate as to which support services new doctors value most highly. Follow-up structured interviews and ethnographic data will help to resolve this issue but were beyond the scope of this study.

Our survey was conducted towards the end of the F1 year. We acknowledge therefore the possibility of recall-bias about the initial level of support offered. However, we believe that doctors will have accurate memories of their initial experiences and feelings as new graduates and would be relatively unlikely to post-rationalise their answers about support. Our results may also be susceptible to non-respondent bias. Comparison of the responses of those who replied early (to the first, second or third survey mailing) with those who replied to one of the subsequent mailings (who would have been non-responders had we not persevered with follow-up mailings) revealed no significant difference in percentages reporting receiving good support (64 % vs 67 % respectively). While this demonstrates that ‘later-response’ bias is unlikely to be a problem, it is possible that some non-responder bias exists which we are unable to quantify. In addition, we note that statistical associations revealed among measured traits in cross-sectional studies such as ours do not prove causality between those traits.

## Conclusion

Government agencies tasked with delivering public health services face the continuing challenge of retaining their staff in order to meet current and future targets [40]. As work-related stress correlates strongly with levels of job satisfaction and morale amongst staff [2, 41], factors that ultimately influence staff retention rates [4, 31], identifying ways to minimise stress and improve job satisfaction for new trainee doctors is critical [27]. This study has revealed that the provision of effective institutional support for new foundation doctors may promote increased job enjoyment and more positive attitudes towards work and thus could help safeguard the long-term retention of junior doctors. In the UK, the responsibility to ensure such support is provided ultimately rests with the Department of Health, the National Health Service (NHS) Trusts and Health Boards, and the LETBs and Deaneries, which must endorse appropriate ongoing support programmes and promote a culture of inclusivity from the time doctors first enter the Foundation Programme.

## Abbreviations

F1, Foundation Training Programme, Year 1; F2, Foundation Training Programme, Year 2; GMC, General Medical Council; LETB, Local Education Training Board; NHS, National Health Service; UK, United Kingdom.

## Additional files

Additional file 1: Table S1. Level of support received by F1 doctors from their Trust compared by sex, ethnicity, graduate status, and choice of Foundation School. Shown are the percentages regarding support as (i) *Very Good*, (ii) *Very Good/Good,* or (iii) *Very Poor/Poor*. (DOC 35 kb) Additional file 2: Figure S1. Percentage of doctors responding to each of the 12 attitude statements on a 5-point scale: Strongly Agree (SA), Agree (A), Neither agree nor disagree (N), Disagree (D), Strongly Disagree (SD). See Methods/Table 1 for full wording of the attitude statements. (TIFF 2294 kb)
