# Supplementary material for: Associations between perceived institutional support, job enjoyment, and intentions to work in the United Kingdom: national questionnaire survey of first year doctors
Source: BMC Med Educ. 2016 May 23;16:151. doi: 10.1186/s12909-016-0673-6 (PMC4878028; doi:10.1186/s12909-016-0673-6)
Supplement: Additional file 1: Table S1. — Level of support received by F1 doctors from their Trust compared by sex, ethnicity, graduate status, and choice of Foundation School. Shown are the percentages regarding support as (i) Very Good, (ii) Very Good/Good, or (iii) Very Poor/Poor. (DOC 35 kb) [file 12909_2016_673_MOESM1_ESM.doc]

**Supplementary Table 1**: Level of support received by F1 doctors from their Trust compared by sex, ethnicity, graduate status, and choice of Foundation School. Shown are the percentages regarding support as (i) *Very Good*, (ii) *Very Good/Good,* or (iii) *Very Poor/Poor*

|  | (i) *Very Good* | (ii) *Very Good/Good* | (iii) ‘*Poor/Very Poor’* |
| --- | --- | --- | --- |
|  | % (N/Total) *^§^* | % (N/Total) *^§^* | % (N/Total) *^§^* |
| (a) Sex |  |  |  |
| Males | 23.8 (202/850) | 62.5 (531/850) | 9.6 (82/850) |
| Females | 23.6 (339/1438) | 65.7 (945/1438) | 7.1 (102/1438) |
| (b) Ethnicity |  |  |  |
| White | 24.2 (409/1687) | 65.9 (1111/1687) | 7.5 (126/1687) |
| Others | 21.4 (121/565) | 60.9 (344/565) | 9.6 (54/565) |
| (c) Graduate status |  |  |  |
| Graduate entrant | 22.3 (79/354) | 63.6 (225/354) | 8.8 (31/354) |
| Non-graduate entrant | 23.9 (451/1890) | 64.7 (1222/1890) | 8.0 (151/1890) |
| (d) 1st choice of Foundation School |  |  |  |
| Got 1^st^ choice | 24.0 (427/1355) | 65.1 (1160/1355) | 8.0 (143/1355) |
| Didn’t get 1^st^ choice | 22.9 (114/498) | 63.3 (315/498) | 7.8 (39/498) |
