# Supplementary figures and images for: Associations between perceived institutional support, job enjoyment, and intentions to work in the United Kingdom: national questionnaire survey of first year doctors
Source: BMC Med Educ. 2016 May 23;16:151. doi: 10.1186/s12909-016-0673-6 (PMC4878028; doi:10.1186/s12909-016-0673-6)

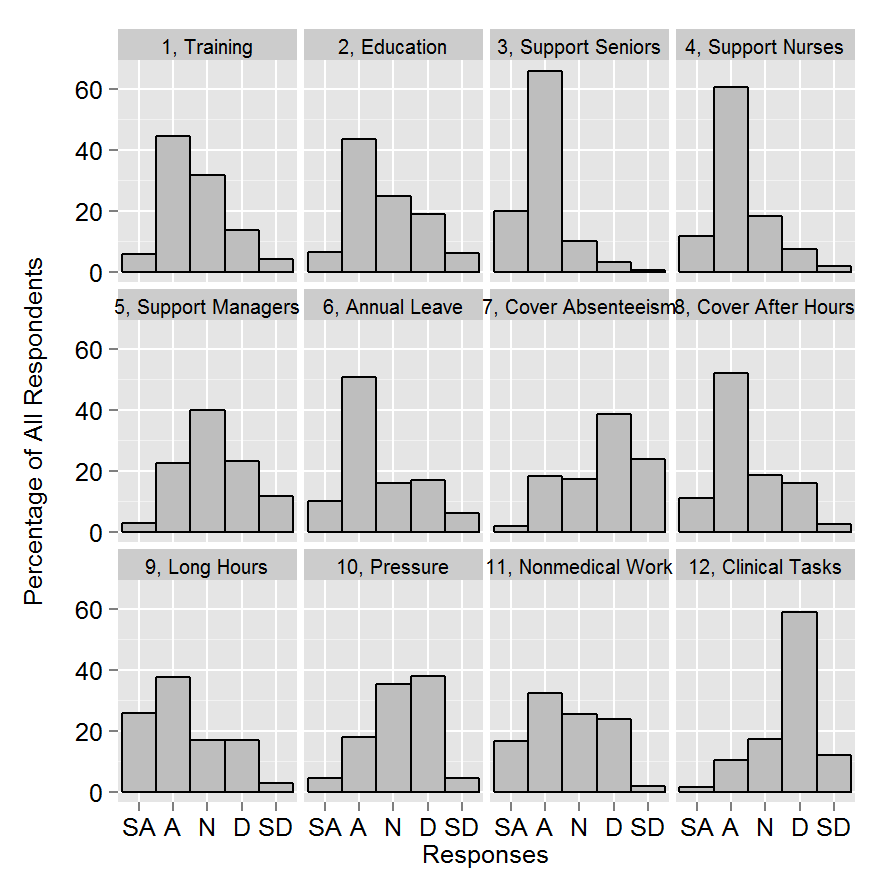

Supplement: Additional file 2: Figure S1. — Percentage of doctors responding to each of the 12 attitude statements on a 5-point scale: Strongly Agree (SA), Agree (A), Neither agree nor disagree (N), Disagree (D), Strongly Disagree (SD). See Methods/Table 1 for full wording of the attitude statements. (TIFF 2294 kb) [file 12909_2016_673_MOESM2_ESM.tiff]
